# Conserved TRAM Domain Functions as an Archaeal Cold Shock Protein via RNA Chaperone Activity

**DOI:** 10.3389/fmicb.2017.01597

**Published:** 2017-08-22

**Authors:** Bo Zhang, Lei Yue, Liguang Zhou, Lei Qi, Jie Li, Xiuzhu Dong

**Affiliations:** ^1^State Key Laboratory of Microbial Resources, Institute of Microbiology, Chinese Academy of Sciences Beijing, China; ^2^School of Life Sciences, University of Chinese Academy of Sciences Beijing, China

**Keywords:** archaea, TRAM, RNA chaperone, cold shock protein, transcriptional antitermination, RNA binding, assist structure RNA degradation

## Abstract

Cold shock proteins (Csps) enable organisms to acclimate to and survive in cold environments and the bacterial CspA family exerts the cold protection via its RNA chaperone activity. However, most Archaea do not contain orthologs to the bacterial *csp*. TRAM, a conserved domain among RNA modification proteins ubiquitously distributed in organisms, occurs as an individual protein in most archaeal phyla and has a structural similarity to Csp proteins, yet its biological functions remain unknown. Through physiological and biochemical studies on four TRAM proteins from a cold adaptive archaeon *Methanolobus psychrophilus* R15, this work demonstrated that TRAM is an archaeal Csp and exhibits RNA chaperone activity. Three TRAM encoding genes (*Mpsy_0643, Mpsy_3043*, and *Mpsy_3066*) exhibited remarkable cold-shock induced transcription and were preferentially translated at lower temperature (18°C), while the fourth (*Mpsy_2002*) was constitutively expressed. They were all able to complement the *cspABGE* mutant of *Escherichia coli* BX04 that does not grow in cold temperatures and showed transcriptional antitermination. TRAM3066 (gene product of *Mpsy_3066*) and TRAM2002 (gene product of *Mpsy_2002*) displayed sequence-non-specific RNA but not DNA binding activity, and TRAM3066 assisted RNases in degradation of structured RNA, thus validating the RNA chaperone activity of TRAMs. Given the chaperone activity, TRAM is predicted to function beyond a Csp.

## Introduction

Cold shock proteins (Csps) are essential for organisms to grow and survive in cold environments. These proteins enable organisms to acclimate to downshifts in temperature by destabilization of cold-induced misfolded RNAs that may cause premature transcription termination, inefficient RNA turnover, and reduced translation. Two categories of bacterial Csps are classified in terms of their working mechanisms, the cold shock protein A (CspA) family of proteins functions mainly as RNA chaperones by affecting transcription or translation of a nascent transcript (Jiang et al., 1997), while the DEAD-box RNA helicases and exoribonuclease unwind and degrade misfolded RNAs under cold temperatures. Unlike RNA helicase, RNA chaperones such as the CspA family of Csps remodel RNAs without a requirement for ATP input (Semrad, 2011). *Escherichia coli* contains nine Csp proteins, among which CspA, CspB, CspG, and CspE are essential for cold acclimation of the bacterium. Typically, the *cspA* gene does not express at mesophilic temperatures but is remarkably induced by cold shock, and the level is reduced after the organism acclimates to the cold (Jiang et al., 1997). Differently, the Csps in *Bacillus* are retained after cold shock (Graumann et al., 1997). *Caulobacter crescentus* also has four Csps: CspA and CspB are cold-induced, and CspC and CspD are induced only in stationary phase (Mazzon et al., 2012). This indicates that the bacterial Csps exert biological roles far beyond cold protection.

The most intensively studied CspA family are basic small proteins of approximately 70 amino acid residues, and the native folds contain an OB (oligonucleotide binding)-fold for binding RNA (Jiang et al., 1997). They accumulate in *E. coli* cells when encountering temperature downshifts and assist the bacterium in acclimation to cold. Through RNA chaperone activity, the CspA family proteins prevent or melt incorrect RNA duplexes formed under lower temperatures, which would hinder efficient translation and regular turnover of a transcript.

Bacterial Csps regulate gene expression at posttranscriptional level. Deletion of *cspE* is shown to affect the transcription of genes containing promoter-proximal elements that induce premature transcription termination. *E. coli* CspC and CspE stabilize the RpoS transcript, a sigma factor that specifically exerts roles under adverse conditions (Shenhar et al., 2012).

However, no Csp has yet been reported for Archaea, although they are abundant in permanently cold environments, including the earth poles and permafrost (Shcherbakova et al., 2016). With the exception of a few psychrophiles and mesophiles (Giaquinto et al., 2007), most Archaea do not contain orthologs to the bacterial *csp*. Transcriptomic analyses of the cold-adaptive methanogenic Archaea *Methanolobus psychrophilus* R15 and *Methanococcoides burtonii* have found that genes encoding small proteins of approximately 70 amino acids that possess a TRAM domain were strongly induced at lower temperatures (Campanaro et al., 2011; Chen et al., 2012). These results suggested that TRAM domain proteins might be involved in the cold adaptation of Archaea. Although there is no sequence homology between TRAM proteins and Csps, they possess the same properties in protein size of approximately 7 kDa, with positively charged amino acids surrounding a surface-exposed aromatic patch, and similar conformation of several antiparallel β-strands containing two RNA-binding motifs. In a recent work, a small protein possessing TRAM domain from a cold-adaptive methanogenic archaeon *Methanococcoides burtonii* was determined to preferentially bind tRNAs and 5S rRNA *in vitro* (Taha et al., 2016), implying its RNA chaperone activity, yet the biological functions of the archaeal TRAM proteins remain to be clarified.

TRAM, an archetype domain, is named after uridine methylase or TRM2 and the adenine thiolation or MiaB families of tRNA-modifying enzymes. These domains are universally distributed in living organisms (Anantharaman et al., 2001). Given that TRAM domains distinctively occur in the proteins that are involved in RNA modification and contain an OB-fold, it is predicted to be an RNA-binding domain. In support of this hypothesis, the TRAM domain in the rRNA methyltransferase RumA binds RNA in protein-RNA co-crystals (Lee et al., 2005). The TRAM domain is also present in proteins associated with translation, such as the IF-2β subunit and ribosomal protein S12 methylthiotransferase RimO (Anton et al., 2008), implying their role in regulation of translation. Interestingly, most Archaea possess a small protein composed of only the TRAM domain (Taha et al., 2016), which we call the TRAM protein.

In this study, through physiological and biochemical studies, we demonstrate that the four single TRAM domain proteins from the cold-adaptive *M. psychrophilus* R15 behave as Csps like the *E. coli* Csps, and that they exhibited RNA chaperone activity.

## Materials and Methods

### Microbial Strains and Culture Conditions

Strains used in this study and their characteristics are listed in Supplementary Table S1. *E. coli* BX04 and plasmid pINIII were kindly provided by Professor Masayori Inouye at the University of Medicine and Dentistry of New Jersey. *E. coli* RL211 was kindly provided by Professor Robert Landick at the University of Wisconsin-Madison. The strains were routinely grown at 37°C in Luria-Bertani (LB) broth.

*Methanolobus psychrophilus* R15 is preserved in our laboratory and cultured at 18°C in mineral medium containing 20 mM trimethylamine, as previously described (Zhang et al., 2008). Growth was monitored by determining the optical density at 600 nm (OD600). For cold shock treatment, the mid-log-phase cultures (OD600 0.41 to 0.43) were pregrown at 18°C or 30°C and incubated at 4°C for 0.5, 1.0, 2.0, and 4.0 h.

### RNA Extraction and qPCR Assay

Three biological replicates of the cold-shocked culture were collected. Five milliliters of each culture was used for RNA extraction using TRIzol as previously described (Li et al., 2015). The extracted RNA was quantified using the NanoDrop ND-100 Spectrophotometer (Gene Company limited, Hong Kong) and analyzed with a 1% agarose gel.

To completely remove the contaminant genomic DNA, 2 μg of each RNA sample was treated with two units of DNase I (Promega, Madison, WI, United States) at 37°C for 5 h. RNA-dependent DNA synthesis was performed using the Moloney murine leukemia virus reverse transcriptase (MMLV RT, Promega) according to the manufacturer’s protocol with random primers (Promega) and 2 μg of DNA-free RNA as the template. The generated cDNA was used as the template for quantitative PCR (qPCR). Real-time qPCR was conducted using the Eppendorf Mastercycler system (Eppendorf, Hamburg, Germany) with 25 μL of SYBR green Premix (TaKaRa) and corresponding primers listed in Supplementary Table S2. The PCR thermal cycle was 95°C for 30 s, followed by 40 cycles of 95°C for 10 s, annealing for 30 s and 72°C for 30 s. Different annealing temperatures were used for the four TRAM genes: 51°C for *Mpsy_0643*, 53°C for *Mpsy_2002*, 59.8°C for *Mpsy_3043*, and 54.3°C for *Mpsy_3066*. Transcript quantification was determined using the comparative threshold cycle (CT) values. To estimate the copy numbers of the transcripts, the expression plasmids pIN-0643, pIN-2002, pIN-3043, and pIN-3066, constructed in the following section, were serially diluted to generate a calibration curve by PCR amplification using the corresponding primer pairs (Supplementary Table S2). The 16S rRNA gene copies were used as a biomass reference. By using the primer pair P3/P4 listed in Supplementary Table S2, almost complete 16S rDNA sequence was amplified and the product was applied as qPCR template to quantify the copy numbers of 16S rRNA using primer pairs P13/P14 and generate a calibration via curve. Quantification of a gene copy number was normalized against the 16S rRNA gene copies and shown as per 10^-4^ 16S rRNA copies.

### Complementation Assay of the Cold-Sensitive *E. coli* BX04 by TRAMs

To test the archaeal TRAMs for cold protection, the open reading frames of the four TRAMs, *Mpsy_0643, Mpsy_2002, Mpsy_3043*, and *Mpsy_3066*, were PCR-amplified using the genomic DNA of *M. psychrophilus* R15 as a template and the corresponding primer pairs (Supplementary Table S2). The PCR products were digested with NdeI/BamHI and cloned into the same sites of pINIII to result in the expression plasmids pIN-0643, pIN-2002, pIN-3043, and pIN-3066, respectively, and they were each transformed into *E. coli* BX04. In parallel, the *E. coli cspE* was cloned and the resulting plasmid pIN-cspE was included as positive control. The empty pINIII plasmid served as a negative control. Overnight cultures of transformants were inoculated into 99 parts of fresh LB medium containing 100 μg/ml ampicillin. When the culture reached an OD600 of 0.9–1.0, 10-fold serial dilutions were prepared, and 6 μL of each dilution was spotted on LB plates containing 100 μg/ml ampicillin, with or without 1 mM isopropyl-D-thiogalactopyranoside (IPTG). The plates were incubated for 2–5 days at 37°C, 22°C and 18°C, respectively.

To examine the expression of TRAM proteins in *E. coli* BX04, IPTG-induced cultures for 2 h at 37°C were pelleted by centrifugation at 12,000 *g* at 4°C for 5 min and lysed by ultrasonication at 240 W using a cycle of 3 s sonication and 3 s pause for 15 min on ice. The total cellular proteins were electrophoresed on Tricine-SDS-PAGE and quantified by Western blot as described below.

### Overexpression and Purification of Proteins

For overexpression of the His6-tagged recombinant proteins, primer pairs P27/P28, P29/P30, P31/P32, and P33/P34 (Supplementary Table S2) were used to amplify *Mpsy_0643, Mpsy_2002, Mpsy_3043*, and *Mpsy_3066*, respectively. After digestion with NcoI/XhoI, the PCR products were cloned into the same sites of the expression plasmid pET28a (Novagen, Madison, WI, United States), resulting in p28a-0643, p28a-2002, p28a-3043, and p28a-3066, respectively. Using the same approach, *E. coli cspA* was cloned into the expression plasmids as p28a-cspA. The pET28a-derived plasmids were each transformed into *E. coli* BL21 (DE3) pLysS (Novagen, Madison, WI, United States). The *E. coli* BL21 (DE3) pLysS strains that each harbors these plasmids were cultivated at 37°C in LB medium containing 50 μg/ml kanamycin. When growth reached an OD600 of 0.4–0.6, 0.5 mM IPTG was added. IPTG-induced cultures were allowed to grow for additional 16 h at 22°C, and cells were harvested from 1 L culture by centrifugation, resuspended in 30 ml of lysis buffer (pH 8.0) containing 20 mM Tris-HCl, 1 M NaCl and 10% glycerol and lysed by ultrasonication at 300 W using a cycle of 3 s sonication and 3 s pause for 30 min on ice. After cell debris was removed by centrifugation, the supernatant was mixed with 0.5% (V/V) polyethyleneimine to remove any contaminated nucleic acids. The mixture was centrifuged at 10,000 *g* for 30 min at the same temperature, and proteins in the supernatant were collected by precipitation with (NH_4_)_2_SO_4_ powder to a final 70% saturation. The protein precipitation was dissolved in 15–20 ml of binding buffer (20 mM Tris-HCl, 100 mM NaCl, and 20 mM imidazole, pH 8.0) and dialyzed overnight at 4°C against the same buffer in 3.5 kDa-pore size dialysis tubing (Spectrumlabs, United States). The dialyzed mixture was centrifuged, and the supernatant was passed through a 0.22-μm filter. The His6-tagged recombinant proteins were purified by passage on a HisTrap HP affinity column (GE Healthcare), followed by HiTrap Q HP anion exchange column (GE Healthcare) and gel filtration chromatography through a Hiload 16/600 Superdex 75 pg column (GE Healthcare). Proteins were concentrated with Amicon Ultrafra-15 concentrators (Millipore) when necessary. The purified proteins were examined by Tricine-SDS-PAGE, and the concentrations were determined using a bicinchoninic acid (BCA) protein concentration assay kit (Pierce, Thermo scientific). The purity of proteins (>99%) and residual nucleic acid were confirmed using a NanoDrop ND-100 Spectrophotometer. To identify the purified proteins, they were digested with trypsin in gels according to the method of Shevchenko et al. (2006) and analyzed by AB Sciex 5800 MALDI-TOF/TOF mass spectrometer.

### Western Blot

A total of 25 ml of each cold-shocked R15 culture, which was cultured for different durations, was harvested at 12,000 *g* at 4°C for 3 min, resuspended in 400 μL PBS buffer (137 mM NaCl, 2.7 mM KCl, 10 mM Na_2_HPO_4_, 2 mM KH_2_PO_4_, pH 7.4), and lysed by ultrasonication at 240 W in a cycle of 3 s sonication and 3 s pause for 15 min on ice. All manipulations were conducted following standard protocols (Sambrook and Russell, 2001) with a few modifications. Total cellular protein was electrophoresed on Tricine-SDS-PAGE below 100 mA for 1.5 h and transferred to a 0.1-μm nitrocellulose filter membrane (Easybio, Beijing, China) under 100 V for 1 h. To fix the proteins to the membrane surface, ddH_2_O and 1 × TBS (2.42 g Tris, 8.0 g NaCl in 1 L, pH 7.6) were used to wash the membrane with gentle shaking for 5 min, and 0.2% glutaraldehyde diluted in 1 × TBS was used to wash the membrane for 45 min. The TRAM3066-specific peptide and TRM2002 total protein-generated antibodies were prepared by the MBL (Beijing Biotech Co., Ltd.). The secondary antibody was purchased from Easybio (Beijing, China).

The density of the Western blotting protein band was determined using Bio-Rad Quantity One (Bio-Rad, Hercules, CA, United States), and the amount of protein was calculated by referring to the gray value of the overexpressed protein.

### RNA Probe Preparation

The Pentaprobes (PP) library developed by Bendak et al. (2012) that comprises twelve 100-bp oligonucleotides encompassing 1,024 possible 5-nucleotide sequence combinations was synthesized by Sangon (Shanghai, China). They were inserted into pUC57 at BamHI/ApaI sites downstream of the T7 promoter, and then used as templates for *in vitro* transcription using the MEGA shortscript T7 Kit (Ambion, Thermo Fisher Scientific) and primer pairs (P35–P47) listed in Supplementary Table S2. The *in vitro* transcription products were purified on 7% urea-PAGE using a ZR small-RNA PAGE Recovery Kit (Zymo Research), and the concentrations were determined using NanoDrop ND-100 Spectrophotometer. All *in vitro* transcribed RNAs were 3′-end biotinylated using a 3′-End Biotinylation Kit (Pierce, Thermo Scientific).

### RNA Electrophoretic Mobility Shift Assay (rEMSA)

An RNA binding assay was performed in a 20-μl reaction mixture containing binding buffer (20 mM Tris-HCl, pH 8.0, 5 mM MgCl_2_, 10 mM KCl, 1 mM DTT, and 10% of glycerol), biotin-labeled RNA substrate, and purified recombinant proteins. After incubation at 25°C for 15 min, the reaction mixtures were loaded onto a 6% polyacrylamide gel and electrophoresed under 100 V for 1 h in 0.5 × TBE running buffer (1 mM EDTA, 45 mM Tris-boric acid, pH 8.0). Free RNA and RNA-protein complexes in gels were transferred to a nylon membrane at 380 mA for 45 min using a Trans-Blot transfer (Bio-Rad Laboratories, Hercules, CA, United States). After cross-linking using a GS Gene Linker^TM^ UV Chamber (Bio-Rad Laboratories, Hercules, CA, United States) for 2 min, a Chemiluminescent Nucleic Acid Detection Module kit (Thermo Scientific) was used to detect chemiluminescence by exposure on a Tanon-5200 Multi instrument (Tanon Science & Technology Co. Ltd., Shanghai, China).

### Surface Plasmon Resonance (SPR) Assay

A surface plasmon resonance (SPR) assay was performed to determine the binding affinities of TRAM3066 and TRAM2002 to ssRNAs on a BIAcore 3000 system (Uppsala, Sweden) as described previously (Li et al., 2010) with minor modifications. The Pentaprobe DNAs with an A_18_C_3_ tail were PCR-amplified by primer pair of P50/P51 in Supplementary Table S2 and then used as the transcription templates for Pentaprobe RNA substrates used in SPR assay. A streptavidin-coated sensor chip SA (Sensor-Chip SA^®^, GE Healthcare) was first conditioned with 3–5 injections (10 ml/min) of 1 M NaCl in 50 mM NaOH until a stable baseline was obtained. The chip cells were washed twice with 0.05% SDS in buffer I (10 mM Tris-HCl, pH 8.0, 300 mM NaCl, 3 mM EDTA, 0.005% Tween20) for 3 min. The biotinylated single-stranded oligonucleotides (SPR linker) were diluted to 10 μM in buffer II (10 mM Tris-HCl, pH 8.0, 300 mM NaCl, 1 mM EDTA) and immobilized on a streptavidin-coated sensor chip SA at a flow rate of 5 ml/min for 10 min. Fifty mM NaOH was injected at 5 μl/min to remove unbound SPR linker until the response units (RU) reached 150. The A_18_C_3_ probe and Pentaprobe RNAs containing A_18_C_3_ tail were immobilized to SPR linkers in different flow cells by manual injection at a flow rate of 1 ml/min until the RU reached a stable state. TRAMs were diluted to 0 to 100 μM by a twofold dilution series with buffer I and injected using the K-inject command at room temperature. CspA and BSA were included as positive and negative controls, respectively. Buffer I (30 ml/min) was used as the running buffer. At the end of each cycle, chip cells were washed with 0.1% SDS to remove bound protein. Data analysis was performed using the BIA evaluation 3.0 program (BIAcore AB, Uppsala, Sweden).

### *In Vivo* Transcription Antitermination Assay

To determine the *in vivo* nucleic acid melting activity of TRAMs, each of *Mpsy_0643, Mpsy_2002, Mpsy_3043*, and *Mpsy_3066* cloned pINIII plasmids was transformed into *E. coli* RL211. The transformants were cultured overnight in LB complemented with 100 μg/ml ampicillin and diluted 100-fold in fresh medium. When the culture grew to an OD600 of 1.0, 6 μl cultures were spotted onto LB plates containing 100 μg/ml ampicillin, with or without 0.2 mM IPTG and 30 μg/ml chloramphenicol, and grown for 2–3 days. *E. coli cspE* and pINIII were used as positive and negative controls, respectively. Expression of TRAM proteins in strain RL211 was determined using the same procedure as in strain BX04.

### Assay of TRAM in Promoting Structured RNA Degradation

The PP6 RNA substrate was labeled with [γ-^32^P] ATP (PerkinElmer, United States) using T4 Polynucleotide Kinase (Thermo Fisher Scientific, United States) according to the manufacturer’s instruction. Radiolabelled PP6 was denatured at 85°C for 5 min and subject to a 30 min temperature-gradient descent till 10°C. 100 fmol of labeled PP6 was mixed with 1,000 pmol TRAM3066 protein in binding buffer (20 mM Tris-HCl buffer, pH 8.0, containing 5 mM MgCl_2_, 10 mM KCl and 1 mM DTT). Various concentrations of RNase A (Ambion, United States) or RNase T1 (Ambion, United States) were added to the PP6-TRAM3066 mixture immediately and incubated at room temperature for 10 min. Ten microliters mixture was loaded onto a 6% or 10% polyacrylamide gel for RNase A and RNase T1 assay, respectively, and electrophoresed at 150 V for 50 min at room temperature. The polyacrylamide gel was vacuum-dried immediately and visualized using autoradiography.

## Results

### Cold Induction of Archaeal TRAM Genes

**FIGURE 1:**
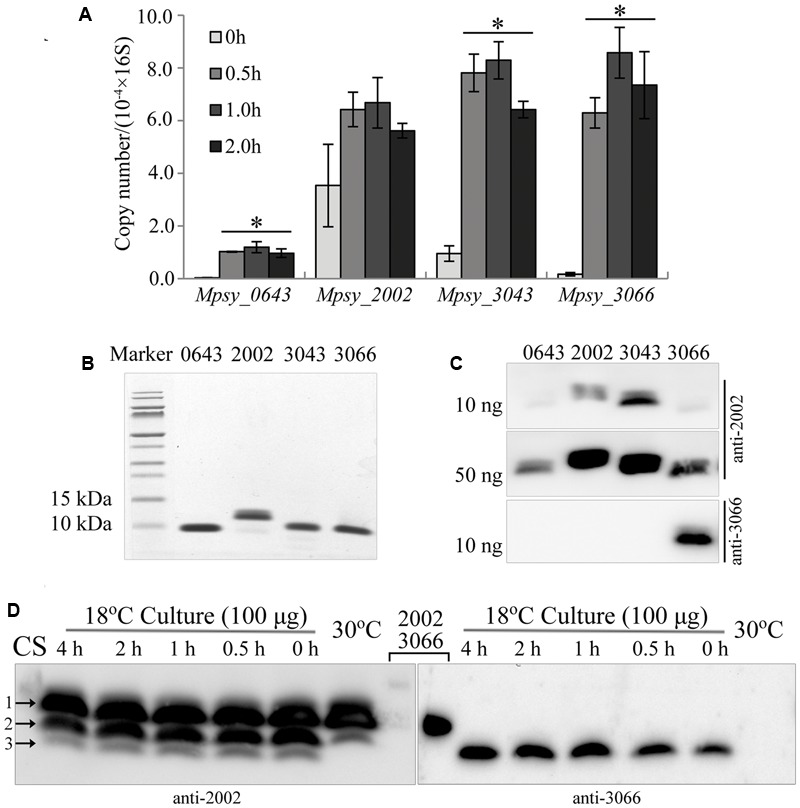
Cold-responsive transcription and translation profiles of the TRAM encoding genes in *Methanolobus psychrophilus* R15. **(A)** Quantitative PCR-determined cold-shock induced transcription of four TRAM genes. Three batches of the middle exponential culture grown at 30°C were 4°C-cold shocked for the indicated time before total RNA was extracted. Using the specific PCR primers listed in Supplementary Table S2 and the PCR thermocycler parameters described in section “Material and Methods,” the cDNA fragment was amplified. The transcript copy number of each TRAM was calibrated against its calibration curve and normalized against the 16S rRNA copies. The mean and standard deviation are shown. ^∗^Statistically significant difference verified by Student’s *t*-test (*P* < 0.05). **(B)** Assays of the purified His6-tagged recombinant TRAM proteins on Tricine-SDS-PAGE. **(C)** Recognition of TRAM2002 and TRAM3066 antibodies to the proteins detected by Western blot. Numbers on the top of gels represent the four TRAMs. **(D)** Cold-responsive translation profiles of the four TRAMs. Each 100 μg of total protein from strain R15 that was grown at 30 and 18°C, and the 4°C-cold shocked 18°C-culture was electrophoresed using a gradient (10–16%) Tricine-SDS-PAGE. The gel was cut in the lane middle that loaded with a mixture of each 10 ng of His6-tagged TRAM2002 and TRAM3066 (lanes 2002 and 3066), and respectively transferred to 0.1-μm nitrocellulose filter membrane. Using the antibodies against TRAM2002 (left) and TRAM3066 (right), Western blot probed the abundance of the four TRAM proteins. The signals of TRAM2002 and TRAM3066 (Lane 2002 and 3066) could only be seen when extend exposure time (data not shown). Arrows indicted TRAM2002 (1), TRAM3043 (2) and a mixture of TRAM 3066 and TRAM0643 (3) by referencing markers and their molecular weights. CS, cold shocked.

*Methanolobus psychrophilus* R15 contains four paralogous genes encoding small TRAM proteins of approximately 7 kDa. Because transcriptomic assay showed that three (*Mpsy_0643, Mpsy_3043*, and *Mpsy_3066*) of four TRAM genes, excluding *Mpsy_2002*, exhibit elevated transcription (Chen et al., 2012) at lower temperatures such as 4°C, cold shock-induced transcription of the four genes was assayed using quantitative RT-PCR (qPCR). Strain R15 was grown at 30°C in trimethylamine until the middle exponential phase and then cold shocked at 4°C for a few hours before harvesting the total RNA. Using the locus-specific PCR primers listed in Supplementary Table S2, transcript levels were quantified by qPCR. Overall, a 9- to 35-fold increase in transcription was detected for *Mpsy_0643, Mpsy_3043*, and *Mpsy_3066* after cold shock for 1 h, whereas Student’s *t*-test did not indicate changed transcription of *Mpsy_2002* in response to temperature decrease (**Figure 1A**). This is consistent with the transcriptomic assay (Chen et al., 2012). Hereafter, the four TRAM proteins are denoted as TRAM0643 (*Mpsy_0643*), TRAM2002 (*Mpsy_2002*), TRAM3043 (*Mpsy_3043*), and TRAM3066 (*Mpsy_3066*).

To detect TRAM protein abundance in cold-shocked cells, we first produced two types of antibodies against TRAM2002 protein and TRAM3066-specific peptide. The antibody raised by TRAM2002 recognized all four TRAMs (**Figure 1B**) but only weakly to TRAM0643 and TRAM3066 (**Figure 1C**). Using this antibody, Western blot detected two proteins in the 30°C culture and three in the 18°C culture (**Figure 1D** and Supplementary Figure S1). By referenced to protein sizes, the two proteins in 30°C culture are predicted to be TRAM2002 (Mw 7.6 kDa) and TRAM3043 (Mw 7.5 kDa) indicated by arrows 1 and 2. The former was constitutively translated, while the latter was induced for approximately 1.7-fold by the temperature downshift. The additional smaller proteins in the 18°C culture can be TRAM0643 and TRAM3066 (Mw 7.2 kDa), as the two appear to be synthesized only at lower temperature. The TRAM3066 antibody specifically recognized its original target, and Western blot confirmed that TRAM3066 was only synthesized in the 18°C culture and not in strain R15 grown at 30°C (**Figure 1D**). Using the purified protein as a reference, the amount of TRAM3066 in the 18°C culture was quantified as less than 5 ng per 100 μg total proteins. While, the density of arrow 3 pointed-band was obviously more intensive than that of 5 ng purified TRAM3066, therefore, we predict this band contained both TRAM0643 and TRAM3066. As the TRAM2002 antibody detected the additional smaller protein(s) in the 18°C culture, both TRAM0643 and TRAM3066 are assumed to be cold-adaptive proteins and reduce translation at higher temperature to a low level below the Western blot detection limit. A few hours of cold shock at 4°C appeared to induce the translation of TRAM3066, but not TRAM0643 (**Figure 1D**). In summary, there are two types of archaeal TRAMs, one represented by TRAM2002 constitutively expresses in response to temperature shift; another type is induced by low temperatures and thus acts as a Csp. Considering the estimated cellular abundance, the constitutively expressed TRAM2002 (0.5% of total cell protein) and cold-induced TRAM3043 (0.1% of total cell protein) (Supplementary Figure S1) can play more important roles; particularly, TRAM2002 may have a function beyond cold protection. While TRAM0643 and TRAM3066 were estimated to comprise approximately 0.02 and 0.004% of the total cell protein (**Figure 1D**), respectively, in the cold and could function mainly as Csps.

Attempt to find whether the different expression modes of the two types of TRAMs are related to the sequences flanked promoter, alignment of the sequences was done for the four TRAM genes. It was found that a CCTW motif located between the promoters and transcription start sites (TSSs) in the three cold-induced TRAMs (Supplementary Figure S2), which is a putative transcription factor (TF) binding site. Another upstream (UP) motif with an AT-rich reverse repeat was found in the promoter upstream regions only in *Mpsy_0643* and *Mpsy_3066*, the two that are only expressed at lower temperatures. However, none of the motifs were found in the *Mpsy_2002* promoter region. This implies that, unlike bacterial *Csps*, expression of archaeal TRAM genes may be regulated at the transcriptional level in response to cold.

### Complementation of the Cold-Sensitive *E. coli* BX04 by Archaeal TRAMs

**FIGURE 2:**
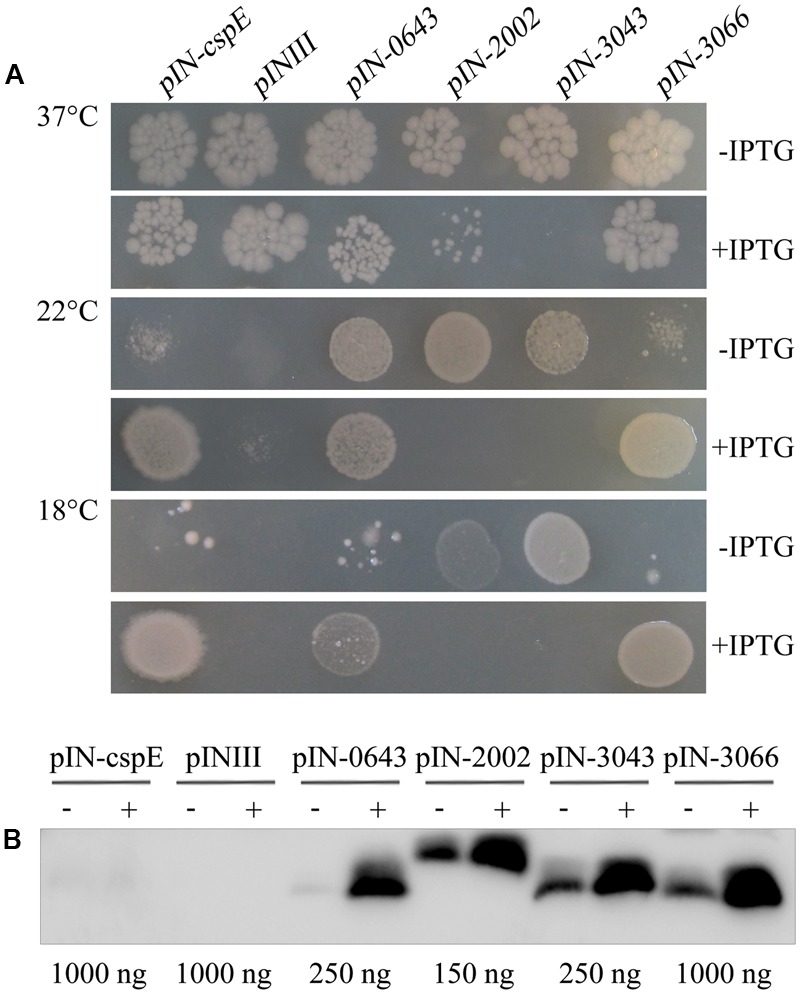
Archaeal TRAMs complemented the cold sensitivity of *Escherichia coli* BX04. **(A)** *E. coli* BX04 carries a quadruple deletion of *cspA, B, G*, and *E* genes, and its growth below 15°C is retarded. Cultures of *E. coli* strains overexpressing each of *Mpsy_0643, Mpsy_2002, Mpsy_3043, Mpsy_3066*, and *cspE* were adjusted to an optical density at 600 nm of 0.9 by dilution with fresh medium. Cultures were serially diluted 10^-5^-fold and spotted onto LB plates supplemented with ampicillin. The plates were incubated at 18, 22, and 37°C for 2–5 days with or without IPTG, as indicated (Supplementary Figure S4A). Growths at 37°C (10^-5^), 22°C (10^-3^), and 18°C (10^-2^) were shown. The pINIII cultures carry the empty plasmid vector. **(B)** Using the antibody against TRAM2002, a Western blot was performed to determine the abundance of four TRAM proteins in *E. coli* BX04. The empty plasmid pINIII and bacterial CspE were included as negative controls. The amount of loaded total protein for each TRAM gene cloned strain is shown beneath the gel. ± on the top of each lane indicates the presence or absence of IPTG.

In addition to cold-induced expression, the archaeal TRAM protein shows a similar tertiary OB-fold structure to the bacterial Csps, possessing five main β-strands (Supplementary Figure S3). To determine whether the structural conservation led to functional conservation, the ability to protect the cold-sensitive *E. coli* strain BX04, which is unable to grow well below 15°C because of a quadruple deletion of the four *csp* genes of *cspA, cspB, cspG*, and *cspE*, was examined. The four TRAM-encoding genes *Mpsy_0643, Mpsy_2002, Mpsy_3043*, and *Mpsy_3066* from *M. psychrophilus* R15 were each cloned into the IPTG-inducible and highly efficient expression vector pINIII. As the CspE-encoding gene from *E. coli*, which was included as a positive control, the four archaeal TRAM genes enabled strain BX04 to grow at low temperature, i.e., 18°C (**Figure 2A** and Supplementary Figure S4A), at which R15 exhibits optimal growth. In contrast, the empty pINIII vector did not allow growth. Differently, higher level of TRAM2002 and TRAM3043 appeared to poison *E. coli* cells; as even at 37°C, *E. coli* BX04 did not grow when expression of the two TRAMs was induced. However, TRAM0643 and TRAM3066 enabled BX04 growing at 18°C only when IPTG-induced to a higher concentration, TRAM0643 (12.4-fold higher protein content) and TRAM3066 (approximately 5.1-fold higher protein content) were more induced by IPTG than the other two proteins (2.1-fold). This highly increased abundance of the two TRAMs did not hurt the *E. coli*, though Western blot detected relatively higher protein content for TRAM0643 than for TRAM2002 and TRAM3043 in BX04 cell (**Figure 2B**) by referenced to the purified four TRAMs (**Figure 1C**). Collectively, the four archaeal TRAMs, with slightly varied behavior between TRAM0643/3043/3066 and TRAM2002, are cold-adaptive proteins similar as the bacterial Csps.

### Archaeal TRAMs as Transcriptional Antiterminators

**FIGURE 3:**
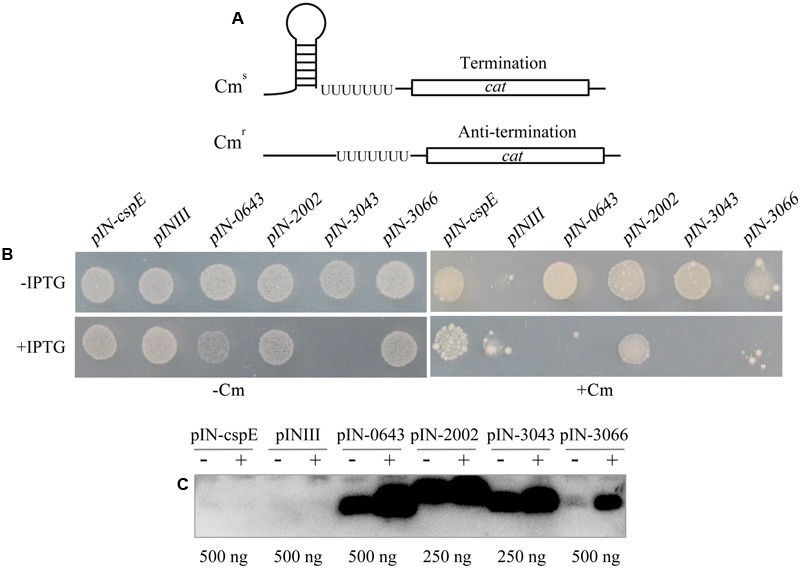
Determination of transcriptional antitermination of TRAM proteins. **(A)** *E. coli* RL211 carrying a *cat* gene cassette positioned downstream of the *trpL* terminator serves as the reporter. Termination (upper figure) and melting of the structured RNA terminator (below figure) allows expression of the chloramphenicol resistance gene *cat*. **(B)** Cells were transformed with pINIII vector alone or pINIII carrying *cspE, Mpsy _0643, Mpsy_2002, Mpsy_3043*, and *Mpsy* _*3066*, as indicated. Cultures were adjusted to an optical density at 600 nm of 1.0 by dilution with fresh medium and 10-fold serially diluted and spotted onto LB plates containing 100 μg/ml ampicillin with or without 0.2 mM IPTG (±IPTG) and 30 μg/ml chloramphenicol (±Cm) as indicated (Supplementary Figure S4B). Growths with Cm (10^0^) and without Cm (10^-3^) were shown. **(C)** Using the antibody against TRAM2002, Western blot was performed to determine the abundance of the four TRAM proteins in *E. coli* RL211. The empty plasmid pINIII and bacterial CspE were included as negative controls. The amount of loaded total protein for each TRAM gene cloned strain is shown beneath the gel. ± on the top of each lane indicates the presence or absence of IPTG.

The primary RNA chaperone activity of the *E. coli* Csp proteins is their transcriptional antitermination of nascent RNAs. To determine whether archaeal TRAMs exhibit the same activity, they were assayed using the procedure of Bae et al. (2000). In this assay, *E. coli* RL211, designed by Landick et al. (1990) was transformed with a pINIII vector carrying each of the IPTG-inducible four archaeal TRAM genes. Strain RL211 carries a *cat* gene immediately downstream of a strong *trpL* transcriptional terminator (**Figure 3A**). In the absence of an RNA chaperone to melt the terminator, *cat* is not expressed, and the cells are sensitive to chloramphenicol. However, if the archaeal TRAMs can melt the RNA structure in the terminator, the downstream *cat* gene is expressed, and the strain becomes resistant to chloramphenicol. Upon transformation with each of the four TRAM encoding genes (*Mpsy_0643, Mpsy_2002, Mpsy_3043*, and *Mpsy_3066*), the RL211 strain became chloramphenicol-resistant in the absence of IPTG (**Figure 3B** and Supplementary Figure S4B), whereas using IPTG induction, only *Mpsy_2002* but not the remaining three TRAM gene-transformed RL211 strains showed chloramphenicol resistance. Similarly, weaker growth as strain BX04 was found for strain RL211 with expression of *Mpsy_0643* and *Mpsy_3043* without chloramphenicol, indicating that the two TRAMs at higher content under IPTG induction impaired *E. coli* RL211 as they did for strain BX04.

Using the TRAM2002 antibody, Western blot determined leaking and IPTG-induced expressions of the four TRAM genes in *E. coli* RL211. As shown in **Figure 3C**, TRAM proteins from the *lpp* promoter promoted leaking expression were estimated at approximately 0.1–0.3% of the total cell protein, which were calibrated based on the Western blot signal intensity of each overexpressed TRAM protein in **Figure 1C**. At this level, the TRAM proteins can exert a role in transcriptional antitermination in *E. coli*. An IPTG-induced elevation of TRAM content will poison *E. coli* RL211, except for TRAM2002, which appears not to be remarkably induced.

### Non-sequence Specificity RNA Binding of Archaeal TRAMs

**FIGURE 4:**
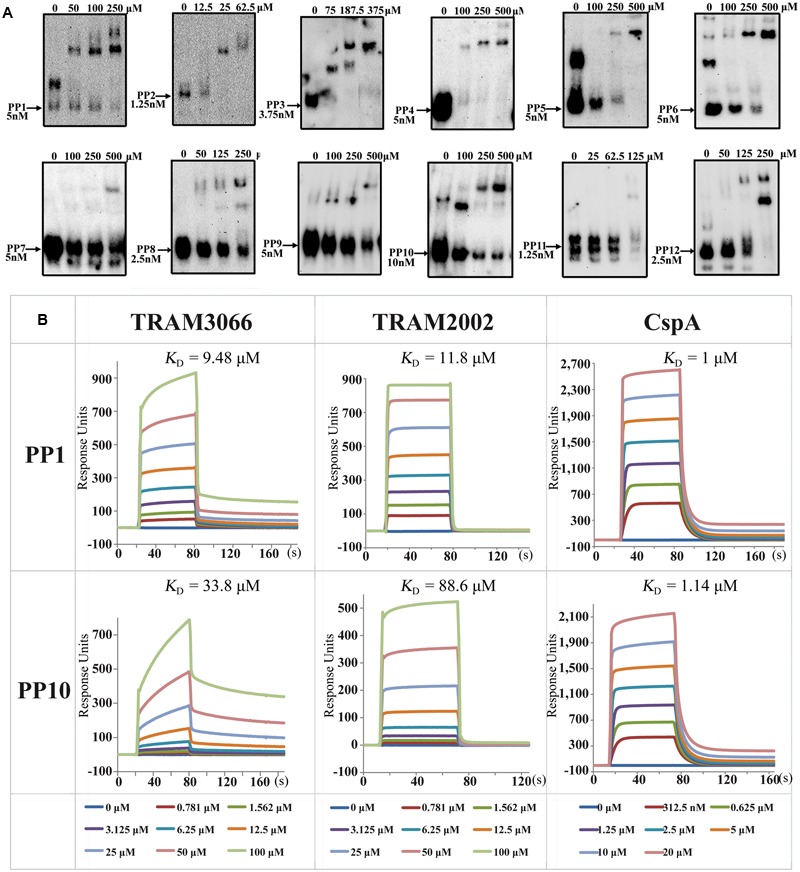
Determination of TRAMs in binding RNA. **(A)** RNA-EMSA (rEMSA) was performed to assay the sequence specificity of TRAM3066-binding RNA. Twelve of the RNA Pentaprobes (PP) (Bendak et al., 2012) were 3′ biotinylated and used as substrates. Purified TRAM3066 was added to the binding mixture at the indicated concentrations. Arrows point to the RNA probe and its concentration. **(B)** Surface plasmon resonance (SPR) assayed the binding affinity of TRAM3066 and TRAM2002 to the selected RNA PP1 and PP10. *E. coli* CspA is included as a reference. Calculated *K*_D_ values are shown in the corresponding diagrams for each pair of protein-RNAs. The protein concentrations for each reaction are color-coded and labeled in the bottom rows. s, second.

The ability of archaeal TRAMs to complement *E. coli* Csp proteins and lead to transcriptional antitermination suggests that they possess activity of RNA chaperone and sequence-non-specific binding to RNA. To directly test this idea, TRAM3066 was selected for a gel retardation RNA-binding assay (rEMSA), and 12 RNA Pentaprobes (PP) which are approximately 100 nt in length and encompass all possible 5 nt RNA sequences (Bendak et al., 2012), were used as binding substrates to investigate the binding specificity of the TRAM. The 3′-end-biotinylated RNA Pentaprobes were incubated with the overexpressed TRAM3066, which bound to most of the 12 PP RNAs, except for PP4, PP7, and PP11, at appropriate ratios of 10,000 to 50,000:1 and selectively formed protein-RNA complexes in a dose-dependent manner (**Figure 4A**). Using the antibody generated from TRAM3066, the RNA-bound protein was confirmed as TRAM3066 (Supplementary Figure S5). These results suggested that TRAM3066 displays almost completely non-specific RNA recognition, which is a characteristic of RNA chaperones. Similar RNA binding was found for His6-tagged TRAM2002, but only faint RNA-protein complex bands appeared in the rEMSA (data not shown). This is most likely attributed to the contaminated RNase degradation of the RNA probe. Though a procedure to remove nucleic acids was used, RNase contamination was observed for all four TRAMs that may explain the much weaker RNA signal in the TRAM3066 complex than the free RNA probe in **Figure 4A**. Co-purification of RNases and TRAM suggests both proteins binding to the same RNA molecules, and adds another clue for TRAM being an RNA-associated protein.

Next, a SPR assay was employed to determine the RNA affinities of the two TRAMs more quantitatively (**Figure 4B**). Low affinities to RNA substrates PP1 and PP10 at *K*_D_ (equilibrium dissociation constant) values ranging from 9 to 34 μM were determined for TRAM3066 and 12 to 89 μM for TRAM2002. These values were comparable but lower than those for CspA under the same condition. However, neither TRAM3066 nor TRAM2002 showed binding to ssDNA or dsDNA even up to a ratio of 10^5^ protein per RNA molecule (data not shown).

### Promotion of Structured RNA Degradation by TRAM3066

**FIGURE 5:**
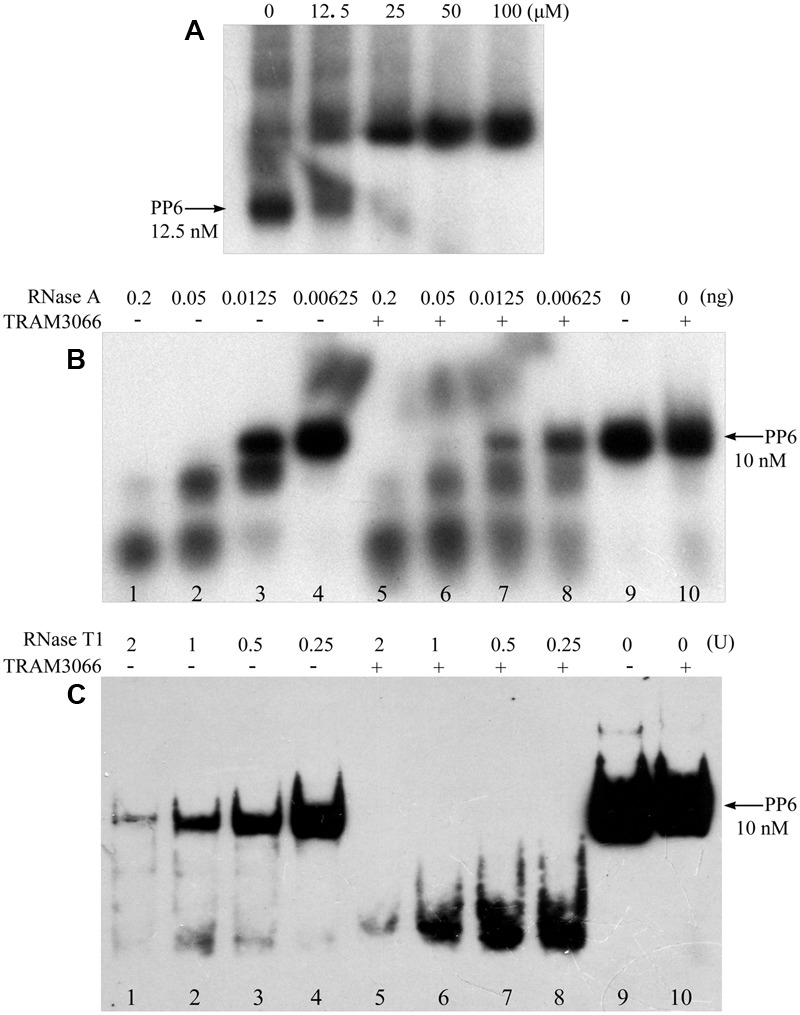
TRAM3066 assisted RNases to hydrolyse structured RNA. The highly structured PP6 RNA was used as the substrate of endonucleases and labeled with [γ -^32^P] ATP. Using the same procedure as in **Figure 4A**, rEMSA was performed to detect the concentration of TRAM3066 shown on the top of the gel binding radiolabelled PP6 RNA **(A)**. Ribonucleolytic assays were performed as described in the Experimental Procedures. PP6 RNA was first mixed with (+) or without (–) 1,000 pmol of TRAM3066 protein, and 0.2, 0.05, 0.0125, and 0.00625 ng (lanes 1–4 and 5–8) of RNase A **(B)** or 2, 1, 0.5, 0.25 U (lanes 1–8) of RNase T1 **(C)** was added. After 10 min of reaction at room temperature, the reaction mixture was electrophoresed on a 6% (RNase A assay) or 10% (RNase T1 assay) acrylamide gel at 150 V at room temperature. Arrows point to full-length PP6 RNA. 0, mock without RNases.

To further validate the RNA duplex melting activity of the archaeal TRAMs, TRAM3066 was selected to test its ability to assist RNase A and RNase T1 degradation of a highly structured RNA PP6, referencing the role of *E. coli* CspA (Jiang et al., 1997). The 92-nt PP6 was 5′-radiolabelled and subjected to 85°C denaturing and 1°C gradient renaturing to 10°C to allow maximal annealing. As shown in **Figure 5A**, rEMSA detected a protein/RNA complex at a ratio of 5,000:1 for protein to RNA. Addition of the equivalent TRAM3066 ratio to the RNase A enzymatic mixture facilitated remarkable degradation of the structured PP6 (**Figure 5B**). For example, neither 6.25 pg of RNase A nor TRAM3066 alone degraded PP6 (**Figure 5B**, Lanes 4 and 10), but addition of TRAM3066 promoted degradation of two-thirds of PP6 by the same amount of RNase A (**Figure 5B**, Lane 8). Similarly, TRAM3066 facilitated RNase T1 to hydrolyse PP6 (**Figure 5C**). This is most likely attributed to TRAM3066 in melting the RNA secondary structure, making it more susceptible to RNase hydrolysis. This confirms the RNA chaperone activity of the archaeal TRAM.

## Discussion

This work, through studies of *in vivo* cold-induced expression and complementation of the cold-growth defect of Csp-deficient *E. coli* BX04, demonstrates that the single-domain TRAM acts as a Csp in methanogenic archaea. Three of the four TRAM genes of *M. psychrophilus* R15 are expressed either only at, or with increased expression upon reduced temperature, e.g., 18°C, and cold shock treatment induced transcription and slightly induced translation. Similar to bacterial Csps, TRAMs exhibit typical RNA chaperone activity in transcriptional antitermination, assisting RNases to degrade structured RNA and binding RNA irrespective of sequence, but with lower affinity. Given the wide distribution of TRAM across archaeal phyla (Taha et al., 2016), we speculate that this family of proteins may function as Csps in the archaeal domain. To elucidate the biological functions beyond cold protection of the archaeal TRAMs, an *in vivo* assay in a genetic tractable archaeal strain will be required.

There appear to be two types of TRAM present in cold-adaptive *M. psychrophilus* R15. TRAM2002 is constitutively expressed in response to temperature shifts and occurs as a housekeeping RNA chaperone, while TRAM0643, TRAM3043, and TRAM3066 are expressed by cold induction and function as cold stress response proteins. Considering the cellular abundance of the four TRAMs, TRAM2002 and TRAM3043 are predictably more important for the archaeon. This is similar to *E. coli*, which possesses two types of Csps: type I (e.g., CspA) rarely presents at 37°C and is highly induced by cold shock, while type II (e.g., CspC) occurs at both temperatures (Thieringer et al., 1998). To probe the molecular basis of the different expression modes of the two types of TRAMs, their 5′ untranslated region (5′UTR) structures at 18 and 30°C were predicted. However, no temperature change-related alternative structures were found for any of the genes (Supplementary Figure S6A). This does not suggest the presence of an “RNA-thermometer” in the 5′UTRs, even though the DNA alignment showed different 5′UTR sequences for the constitutively expressed *Mpsy_2002* and the other three genes (Supplementary Figure S6B). Moreover, the cellular mRNA half-life of TRAM3066 was almost the same in cold adaptive and mesophilic cultures (data not shown). Whereas, there appears to be consensus motifs present proximately to the promoters in the three cold-induced TRAMs (Supplementary Figure S2). Therefore, it is unlikely that posttranscriptional regulation, which is for the bacterial *csp*s, is involved in control temperature-responsive expression of methanogenic TRAMs, rather than at the transcriptional level in response to cold.

Though similar to the bacterial Csps in RNA chaperone activity, the archaeal TRAMs appear to have a lower RNA affinity than CspA determined in both rEMSA and SPR assays (**Figure 4**). It was determined that six residues, Trp^11^, Phe^18^, Phe^20^, Phe^31^, His^33^, and Phe^34^ in CspA and CspE are key to the RNA binding activity (Supplementary Figure S3) (Newkirk et al., 1994; Feng et al., 1998), and Phe^18^, Phe^31^, and His^33^ are key for RNA melting (Phadtare et al., 2002). While TRAM only contains four aromatic residues analogy to the six RNA binding residues of CspA, this may be the reason of the lower RNA affinity. Protein superimposition of TRAM3066 and CspA showed that the C-terminus half fragment of TRAM is well matched to N-terminus half fragment of CspA (Supplementary Figure S3), and two of four aromatic residues of TRAM match the two CspA residues for RNA melting, thus explaining the molecular basis of TRAM in promoting structured RNA degradation (**Figure 5**).

In addition to cold protection, bacterial Csps display additional functions such as antioxidative stress, stationary growth and anti-antibiotic activity (Chanda et al., 2009). Given their RNA chaperone activity, the biological roles of TRAM are also predicted to extend beyond Csp functions, such as the posttranscriptional modulator CspC, which is involved in global gene regulation in *E. coli* (Horn et al., 2007). In our unpublished work, deletion of the only TRAM gene in *Methanococcus maripaludis* remarkably retarded the growth. Further study of the working mechanisms of TRAM is ongoing.

Because TRAM is a conserved domain among RNA modification proteins that are ubiquitously distributed in organisms, the findings in this work would shed light on understanding the TRAM domain in rRNA and ribosomal protein methylation in other organisms.

## Author Contributions

XD and JL designed the study. BZ, LY, LZ, LQ and JL performed experiments. XD, JL, and BZ analyzed data. XD and BZ wrote the manuscript.

## Conflict of Interest Statement

The authors declare that the research was conducted in the absence of any commercial or financial relationships that could be construed as a potential conflict of interest.
