# Supplementary material for: Conserved TRAM Domain Functions as an Archaeal Cold Shock Protein via RNA Chaperone Activity
Source: Front Microbiol. 2017 Aug 22;8:1597. doi: 10.3389/fmicb.2017.01597 (PMC5572242; doi:10.3389/fmicb.2017.01597)
Supplement: Supplementary file 1 [file Data_Sheet_1.docx]

**Conserved TRAM domain functions as an archaeal cold shock protein via RNA chaperone activity**

Bo Zhang^1,2^, Lei Yue ^1,2^, Liguang Zhou ^1,3^, Lei Qi ^1,2^, Jie Li ^1,2^*, Xiuzhu Dong ^1,2^*

Supplementary information on line

Supplementary Figures

**
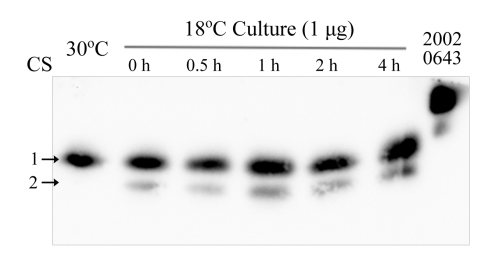
**

**Supplementary Figure S1. Cold-responsive translation profiles of the four** **TRAM genes in *Methanolobus psychrophilus* R15**. Each 1 μg of the total protein from strain R15 that grown respectively at 30°C and 18°C, and the 4°C-cold shocked 18°C-culture was electrophoresed on a gradient (10-16%) Tricine-SDS-PAGE. Using an antibody against TRAM2002, western blot probed the cellular abundances of the four TRAM proteins. A mixture of each 10 ng of His_6_-tagged TRAM2002 and TRAM0643 proteins were included as the protein markers. Arrows indicted TRAM2002 (1), TRAM3043 (2) by referencing markers and their molecular weights. CS, cold shocked.


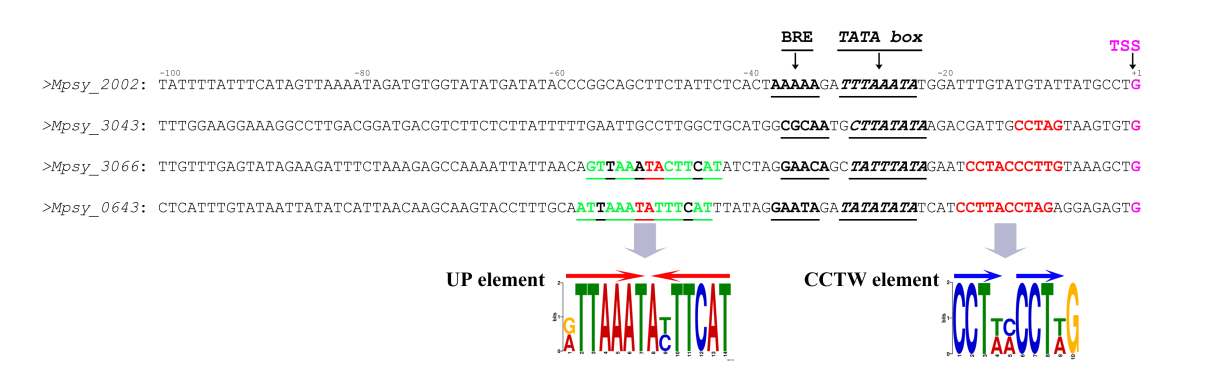


**Supplementary Figure S2. Analysis of the putative regulation elements that may be involved in the promoter upstream region of the four archaeal TRAM genes.** A scheme shows the two motifs respectively upstream and downstream the promoter (underlined) searched by MEME program. TSS: transcription start site; BRE: transcription factor B recognition element.

**
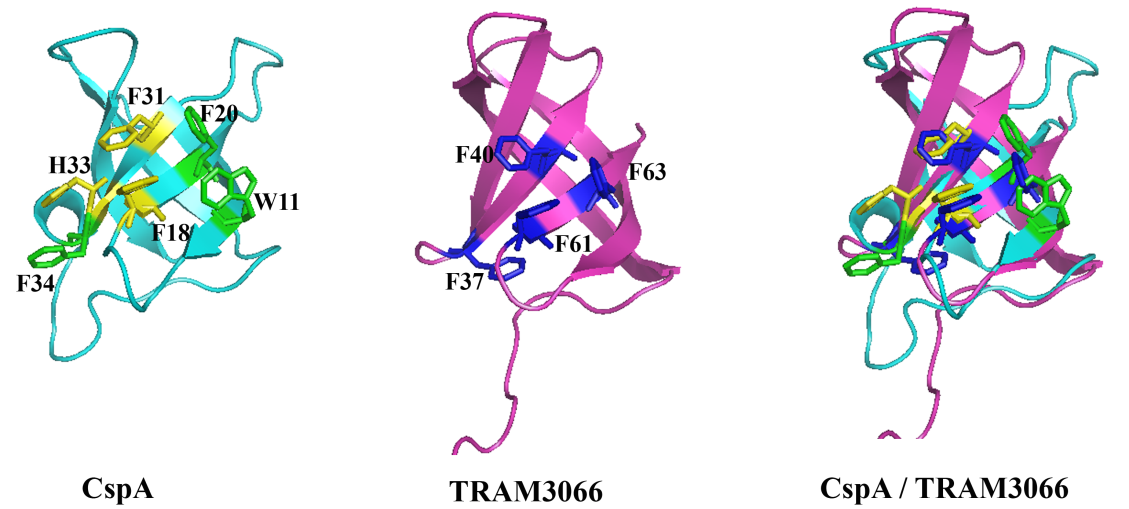
**

**Supplementary Figure S3.** **Comparison of protein structures of the *E. coli* CspA and the archaeal TRAM3066.** Left panel, structure of the *E. coli* CspA shows the six key residues for RNA binding and the three key residues (yellow) for RNA melting. Middle panel, the homologous modeling of TRAM3066 is based on the structure of MM1357 from *Methanosarcina mazei* and shows four aromatic residues (blue) that are predicted to responsible for RNA binding. Right panel, superimposition of TRAM3066 over CspA shows that F36 and F40 of TRAM are well matched the two CspA melting residues F18 and F31.


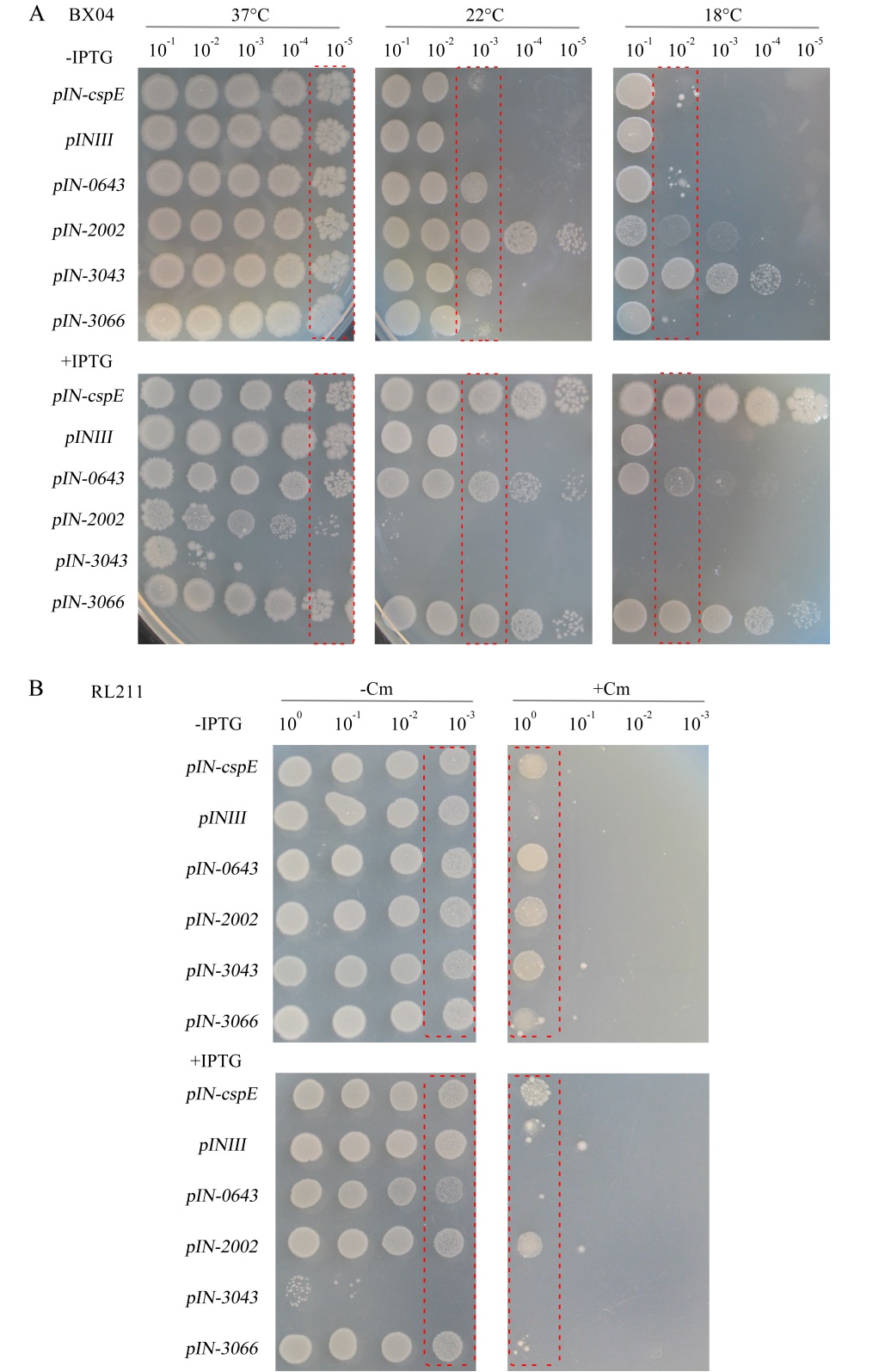


**Supplementary Figure S4. Assays of Archaeal TRAMs in complementing the cold sensitivity of *E. coli* BX04 (A) and transcriptional antitermination (B).** Overnight liquid cultures of *E. coli* strains overexpressing each of *Mpsy_0643*, *Mpsy_2002*, *Mpsy_3043*, *Mpsy_3066*, and *cspE* were inoculated into fresh medium. When the culture reached an OD600 of 0.9-1.0, they were adjusted to an optical density at 600 nm of 0.9 with fresh medium. Cultures were 10-fold diluted and spotted onto LB plates with 100 μg/ml ampicillin supplemented. (A) Plates were incubated at 37°C, 22°C and 18°C for 2-5 days with or without IPTG, as indicated and framed spots of dilution are shown in Figure 2. (B) Plates were incubated at 37°C for 2-3 days with or without 0.2 mM IPTG (+/-IPTG) and 30 μg/ml chloramphenicol (+/-Cm), respectively. Framed spots of dilution are shown in Figure 3.


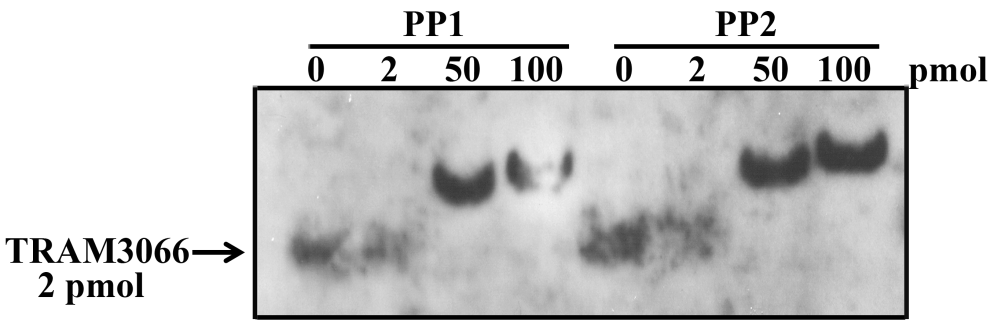


**Supplementary Figure S5. Western blot assay of TRAM3066 in the protein-RNA complexes.** An EMSA assay was performed used the same procedure as in Figure 4 except that a fixed amount of TRAM3066 (2 pmol) was mixed with 0, 2 pmol, 50 pmol, 100 pmol PP1 RNA or PP2 RNA, respectively. The mixtures were electrophoresed on an 8% polyacrylamide gel at 100 V for 120 min at room temperature and then transferred to a 0.1-μm nitrocellulose membrane under 400 mA for 25 min. By using the antibody generated from TRAM3066, Western blotting was performed using the procedure as described in Materials and Methods.

**
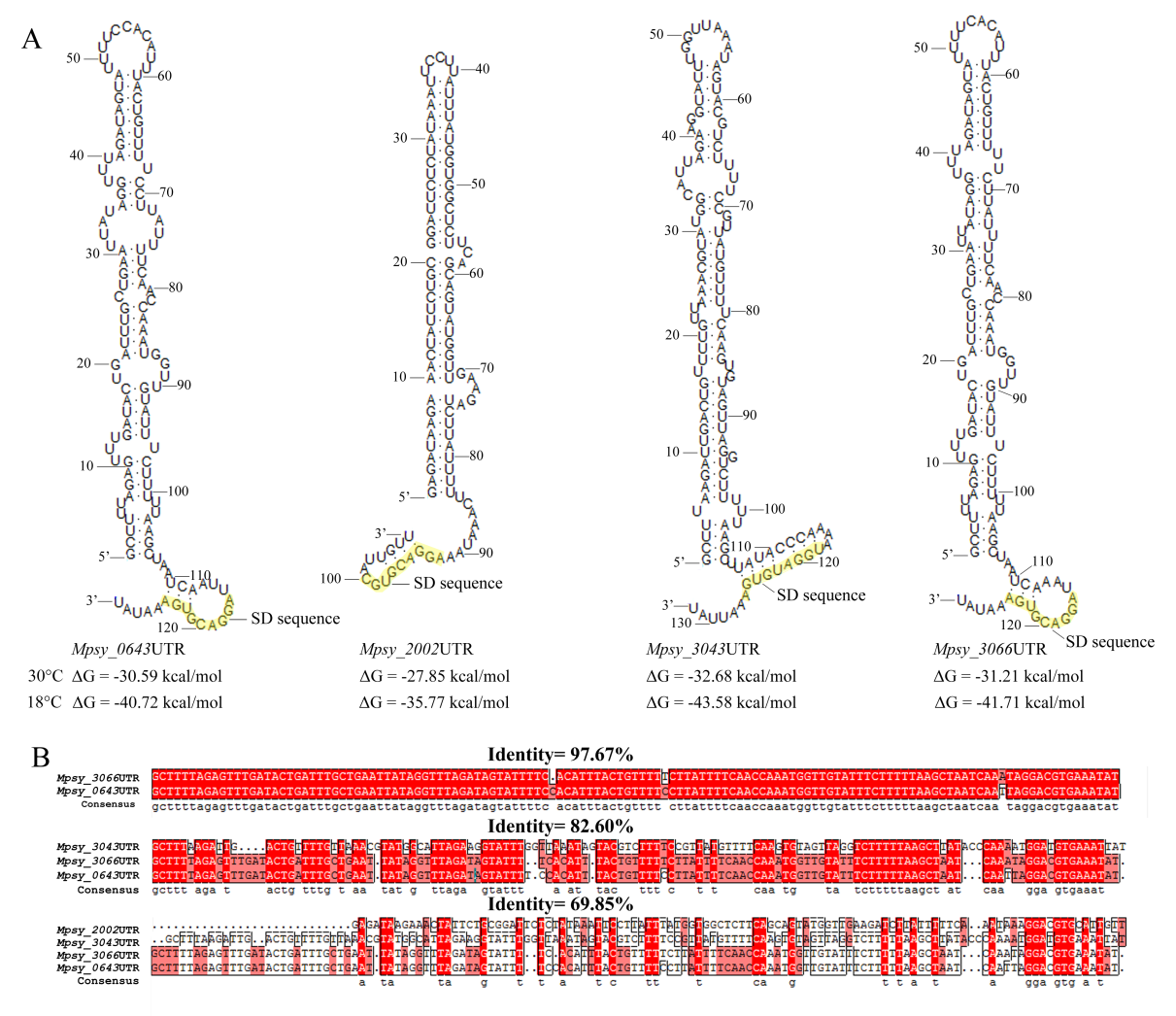
**

**Supplementary Figure S6. Analysis of the putative regulation elements that may be involved in the 5**′ **untranslated region (5**′**UTR) and the promoter upstream region of the four archaeal TRAM genes.** (A) 5′UTR sequences including the regions from dRNA-seq determined transcription start sites (TSS) to immediately upstream of the start codons (Li et al., 2015) were predicted for RNA structures using the mfold Web Server (http://unafold.rna.albany.edu/?q=mfold). Yellow shadowed bases are the predicted SD sequences. (B) DNA sequences of the four TRAMs 5′UTRs were aligned for homolog analysis. Red shadowed the conserved bases.

Supplementary tables

**Table S1. Strains and plasmids used in this study**

| **Strains and plasmids** | **Characteristics and descriptions** | | **Reference or sources** | | |
| --- | --- | --- | --- | --- | --- |
| **Strains** |  | |  | | |
| *E. coli* DH5α | F^-^φ80d *lac*ZΔM15 Δ(*lac*ZYA-*arg* F) U169 *end*A1 *rec*A1 *hsd*R17(r_k_^-^,m_k_^+^) *sup*E44λ- *thi* -1 *gyr*A96 *rel*A1 *pho*A | Transgene, Beijing | |  |  |
| *E. coli* BL21 (DE3) pLysS | F^-^ ompT hsdS_B_(r_B_ - m_B_ -) gal dcm(DE3)pLysS Cam^r^ | | Novagen, Madison | |  |
| *E. coli* BX04 | Four csp genes (*cspA*,*cspB*,*cspE*, *cspG*) deleted | | Xia et al., 2001 | |  |
| *E. coli* RL211 | ρ-independent *trp*L terminator followed by *cat* gene | | Landick et al., 1990 | |  |
| *Methanolobus psychrophilus* R15 | Grows optimally at 18°C | | Zhang et al. 2008 | |  |
| **Plasmids** |  | |  | | |
| pINIII | Amp^R^ | | Xia et al., 2001 | | |
| pIN-cspE | pINIII with *E. coli cspE* inserted between NdeI and BamHI, Amp^R^ | | This study | | |
| pIN-0643 | pINIII with *Mpsy_0643* inserted between NdeI and BamHI, Amp^R^ | | This study | | |
| pIN-2002 | pINIII with *Mpsy_2002* inserted between NdeI and BamHI, Amp^R^ | | This study | | |
| pIN-3043 | pINIII with *Mpsy_3043* inserted between NdeI and BamHI, Amp^R^ | | This study | | |
| pIN-3066 | pINIII with *Mpsy_3066* inserted between NdeI and BamHI, Amp^R^ | | This study | | |
| pET28a | Kan^R^ | | Novagen, Madison | | |
| p28a-0643 | pET28a with *Mpsy_0643* inserted between NcoI and XhoI, Kan^R^ | | This study | | |
| p28a-2002 | pET28a with *Mpsy_2002* inserted between NcoI and XhoI, Kan^R^ | | This study | | |
| p28a-3043 | pET28a with *Mpsy_3043* inserted between NcoI and XhoI, Kan^R^ | | This study | | |
| p28a-3066 | pET28a with *Mpsy_3066* inserted between NcoI and XhoI, Kan^R^ | | This study | | |
| p28a-cspA | pET28a with *E. coli cspA* inserted between NcoI and XhoI, Kan^R^ | | This study | | |
|  | | | | | |

**Table S2. Primers or probes used in this study**

| **Primers** | **Sequences(5’-3’)** | **Purposes** |
| --- | --- | --- |
| P1 | GATAACTACGATACGGGAGGG | P1/P2 for qRT-PCR standard curve of *Mpsy_0643*, *Mpsy_2002*, *Mpsy_3043*, *Mpsy_3066* |
| P2 | CGGACCAAATAACCCGCTA |  |
| P3 | GCGAGTCAAATGTTCTTC | P3/P4 for qRT-PCR standard curve of 16S rDNA |
| P4 | CTACGGCTACCTTGTTAC |  |
| P5 | AATTCAACTGCTCCAGTAGAAGCTGGAATA | P5/P6 qRT-PCR of *Mpsy_0643* |
| P6 | GCGGCACTCATAACTTTGTTAACTGTGATA |  |
| P7 | TCCTGTAGCTGCTGGAGAAACA | P7/P8 qRT-PCR of *Mpsy_2002* |
| P8 | TGGTGATCTTGATGTTTACAGTC |  |
| P9 | GCCGATGTTCAACAGAACTGAAGAAACC | P9/P10 qRT-PCR of *Mpsy_3043* |
| P10 | AACTTCGTCGCCAACCTTGGTGCCT |  |
| P11 | CAGTCAAGTGCTCCAGTAGAAGCTGGACAG | P11/P12 qRT-PCR of *Mpsy_3066* |
| P12 | TACCTTATTGACCTTTATCGTG |  |
| P13 | ATAGATGCTGGAATGCTCTG | P13/P14 qRT-PCR of 16S rDNA |
| P14 | GGCTCTTGCTCTCACAAC |  |
| P15 | GGAATTCCATATGTCTAAGATTAAAG | P15/P16- pIN-cspE |
| P16 | CGCGGATCCTTACAGAGCGATTA |  |
| P17 | GGAATTCCATATGTTTAGTAACATGAATTCAACTGCTCCA | P17/P18- pIN-0643 |
| P18 | CGCGGATCCTTAAGCAACT |  |
| P19 | GGAATTCCATATGTTTAACAATA | P19/P20- pIN-2002 |
| P20 | CGCGGATCCTTATTCTTCCTTTTC |  |
| P21 | GGAATTCCATATGTTCAACAGA | P21/P22- pIN-3043 |
| P22 | CGCGGATCCTTATTCTGCAACT |  |
| P23 | GGAATTCCATATGTTTAGTAACATGCAGTCAAGTGCTCCAGTAG | P23/P24- pIN-3066 |
| P24 | CGGGATCCTTAAGCAACTTCGCCAAA |  |
| P25 | CATGCCATGGTGTCCGGTAAAATGACTG | P25/P26- p28a-cspA |
| P26 | CCGCTCGAGCAGGCTGGTTACGTTA |  |
| P27 | CATGCCATGGTGTTTAGTAACATGAATTCAACTGCTCCA | P27/P28- p28a-0643 |
| P28 | CCGCTCGAGAGCAACTTCGCCAAAA |  |
| P29 | CATGCCATGGTGTTTAACAATAATCAAGAGTC | P29/P30- p28a-2002 |
| P30 | CCGCTCGAGTTCTTCCTTTTCTGCA |  |
| P31 | CATGCCATGGTGTTCAACAGAACTGA | P31/P32- p28a-3043 |
| P32 | CCGCTCGAGTTCTGCAACTTGG |  |
| P33 | CATGCCATGGTGTTTAGTAACATGCAGTCAAGTGCTCCAG | P33/P34- p28a-3066 |
| P34 | CCGCTCGAGAGCAACTTCGCCAAAA |  |
| P35 | CCTCTTCGCTATTAC | general forward primer |
| P36 | TAAAGTAAGATAAGGCAAGACAAG | P35/P36 for PP1 template |
| P37 | TAGAATAGAGCGCAACTGAAGTCA | P35/P37 for PP2 template |
| P38 | ATGGGTAGGATAGGCGAGGC | P35/P38 for PP3 template |
| P39 | GGTGGGATTAGGGCAGCTCGAC | P35/P39 for PP4 template |
| P40 | CTCCAACCTCACACCAC | P35/P40 for PP5 template |
| P41 | CGGGATCCCGTACCATACTATAC | P35/P41 for PP6 template |
| P42 | CGGAATTCTACGA | P35/P42 for PP7 template |
| P43 | TATCTTACTTTAGTTTCATT | P35/P43 for PP8 template |
| P44 | CGCTCTATTCTACTGT | P35/P44 for PP9 template |
| P45 | TATCCTACCCATTG | P35/P45 for PP10 template |
| P46 | CCTAATCCCACCTA | P35/P46 for PP11 template |
| P47 | GTGAGGTTGGAGTCC | P35/P47 for PP12 template |
| P48 | 5’Bio-/rU/GGGTTTTTTTTTTTTTTTTTT | P48 used as the SPR linker (Bendak et al., 2012) |
| P49 | AAAAAAAAAAAAAAAAAACCC | P49 used as the SPR probe |
| P50 | TGTAAAACGACGGCCAGTGA | P50/P51 for PP1 and PP10 DNA templates containing A_18_C_3_ sequence |
| P51 | GCTATGACCATGATTACGCCAAG |  |

Reference:

Bendak, K., Loughlin, F.E., Cheung, V., O'Connell, M.R., Crossley, M. and Mackay, J.P. (2012) A rapid method for assessing the RNA-binding potential of a protein. *Nucleic Acids Res.* 40, e105. doi: 10.1093/nar/gks285

Landick, R., Stewart, J. and Lee, D.N. (1990) Amino acid changes in conserved regions of the beta-subunit of *Escherichia coli* RNA polymerase alter transcription pausing and termination. *Gene Dev.* 4, 1623-1636. doi: 10.1101/Gad.4.9.1623

Li, J., Qi, L., Guo, Y., Yue, L., Li, Y.P., Ge, W.Z. et al. (2015) Global mapping transcriptional start sites revealed both transcriptional and post-transcriptional regulation of cold adaptation in the methanogenic archaeon *Methanolobus psychrophilus*. *Sci. Rep.* 5, srep 9209. doi: 10.1038/srep09209

Xia, B., Ke, H.P. and Inouye, M. (2001) Acquirement of cold sensitivity by quadruple deletion of the cspA family and its suppression by PNPase S1 domain in *Escherichia coli*. *Mol. Microbiol.* 40, 179-188. doi: 10.1046/j.1365-2958. 2001.02372.x

Zhang, G.S., Jiang, N., Liu, X.L. and Dong, X.Z. (2008) Methanogenesis from methanol at low temperatures by a novel psychrophilic methanogen, "*Methanolobus psychrophilus*" sp. nov., prevalent in Zoige wetland of the Tibetan plateau. *Appl. Environ. Microbiol.* 74, 6114-6120. doi: 10.1128 /AEM.01146-08
